# Publisher Correction: Cryo-EM structure of a RAS/RAF recruitment complex

**DOI:** 10.1038/s41467-023-41365-9

**Published:** 2023-09-07

**Authors:** Eunyoung Park, Shaun Rawson, Anna Schmoker, Byeong-Won Kim, Sehee Oh, Kangkang Song, Hyesung Jeon, Michael J. Eck

**Affiliations:** 1 Department of Cancer Biology, Dana-Farber Cancer Institute, Boston, MA 02215 USA; 2 Department of Biological Chemistry and Molecular Pharmacology, Harvard Medical School, Boston, MA 02115 USA; 3 Department of Biochemistry & Molecular Biotechnology, University of Massachusetts Chan Medical School, 364 Plantation St, Worcester, MA 01605 USA; 4 Present Address: Pfizer R&D Center, 3200 Walnut St, Boulder, CO 80301 USA; 5 Present Address: New Drug Development Center, Osong Medical Innovation Foundation, Cheongju, Chungbuk 28160 Republic of Korea

**Keywords:** Cryoelectron microscopy, Oncogenes, Kinases

Correction to: *Nature Communications* 10.1038/s41467-023-40299-6, published online 29 July 2023

The original version of this Article contained errors in Table 1. The correct version of the final row of the 2nd column states ‘127.3 ± 77.2’ instead of the original, incorrect ‘±77.2’. The correct version of the final column of the 2nd row states ‘21.2 (ref. 24) 356 (ref. 17)’ instead of the original, incorrect ‘21.2 (ref. 24, 356 (ref. 17)’; the correct version of the final column of the 3rd row states ‘190.5 (ref. 24) 152 (ref. 17)’ instead of the original, incorrect ‘190.5 (ref. 24, 152 (ref. 17)’.This has been corrected in both the PDF and HTML versions of the Article.

