# Supplementary material for: Cryo-EM structure of a RAS/RAF recruitment complex
Source: Nat Commun. 2023 Jul 29;14:4580. doi: 10.1038/s41467-023-40299-6 (PMC10387098; doi:10.1038/s41467-023-40299-6)
Supplement: Supplementary file 1 — Supplementary Information [file 41467_2023_40299_MOESM1_ESM.pdf]

Supplementary Information

Cryo-EM structure of a RAS/RAF recruitment complex

Eunyoung Park[◊], Shaun Rawson[◊], Anna Schmoker, Byeong-Won Kim, Sehee Oh, Kangkang Song, Hyesung Jeon*, Michael Eck*

[◊] These authors contributed equally.
* Corresponding Authors: Michael J. Eck eck@crystal.harvard.edu
Hyesung Jeon hjeon@crystal.harvard.edu

Supplementary Information:

Supplementary Table 1
Supplementary Figures 1-10
Supplementary references

Supplementary Table 1. Cryo-EM data collection, refinement and validation statistics.

	KRAS-Up (EMD-27428) (PDB 8DGS)	KRAS-Front (EMD-27429) (PDB 8DGT)	Δ155-BRAF/MEK/14-3-3 (EMD-40253)
**Data collection and processing**			
Magnification	105,000 x	105,000 x	130,000 x
Voltage (kV)	300	300	300
Electron exposure (e–/Å^2)	45.6	54	~50
Defocus range (μm)	-1.8 – -2.8	-1.5 – -2.5	-0.2 – -1.2
Pixel size (Å)	0.85	0.825 (1.03)	0.53 (1.06)
Symmetry imposed	C1	C1	C1
Initial particle images (no.)	570,743	3,989,095	536,128
Final particle images (no.)	69,377	190,489	91,490
Map resolution (Å)	4.3	3.9	4.1
0.143 FSC threshold			
**Refinement**			
Initial model used (PDB code)	6NYB, 6VJJ	6NYB, 6VJJ	
Map sharpening *B* factor (Å^2)	-252		
Model composition			
Non-hydrogen atoms	11089	11097	
Protein residues	1368	1367	
Ligands	4	4	
Metals	4	4	
B factors (Å^2)			
Protein	206.91	86.66	
Ligand	224.27	62.62	
R.m.s. deviations			
Bond lengths (Å)	0.004	0.006	
Bond angles (°)	0.631	1.136	
Validation			
MolProbity score	2.03	2.05	
Clashscore	10.61	9.92	
Poor rotamers (%)	3.73	2.98	
Ramachandran plot			
Favored (%)	97.76	97.02	
Allowed (%)	2.24	2.76	
Disallowed (%)	0.0	0.22	
Model vs Data			
CC (mask)	0.66	0.51	
CC (box)	0.79	0.55	
CC (peaks)	0.59	0.47	
CC (volume)	0.65	0.52	
Mean CC for ligands	0.72	0.64	

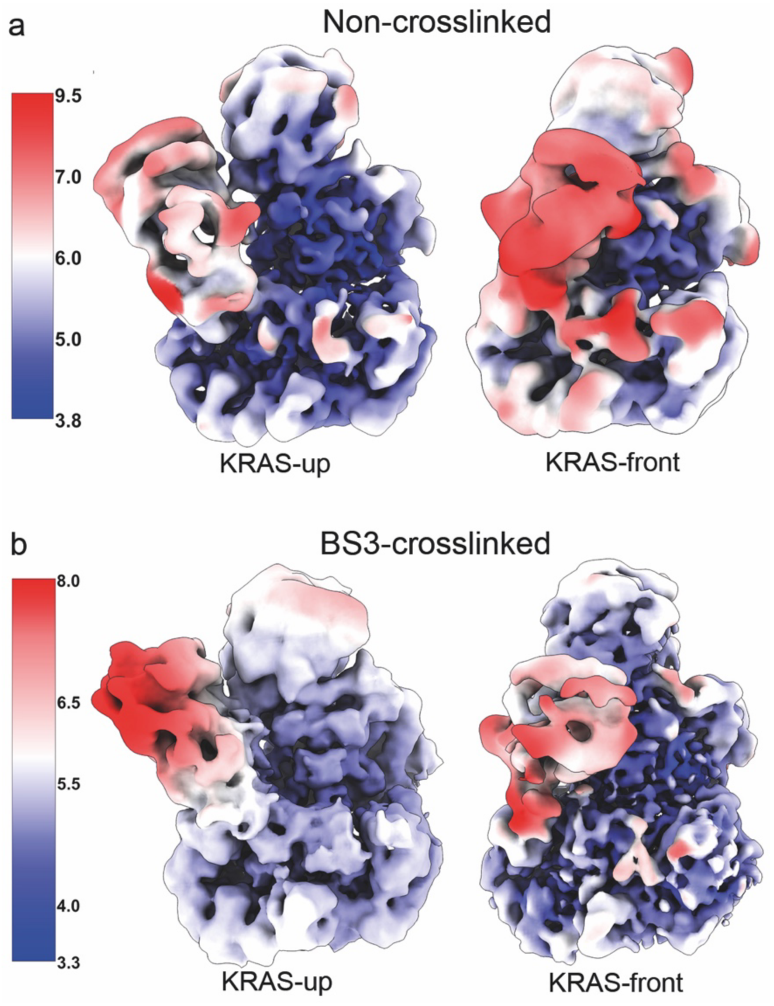

Supplementary Figure 1. Cryo-EM density maps obtained from single particle reconstructions from crosslinked and non-crosslinked KRAS/BRAF/MEK1/14-3-3 samples. 3D classification of particles allowed identification of two conformations of the complex, KRAS-up and KRAS-front, in both non-crosslinked (a) and BS3-crosslinked (b) samples. Maps are colored by resolution (Å) as indicated by the bars on the left.

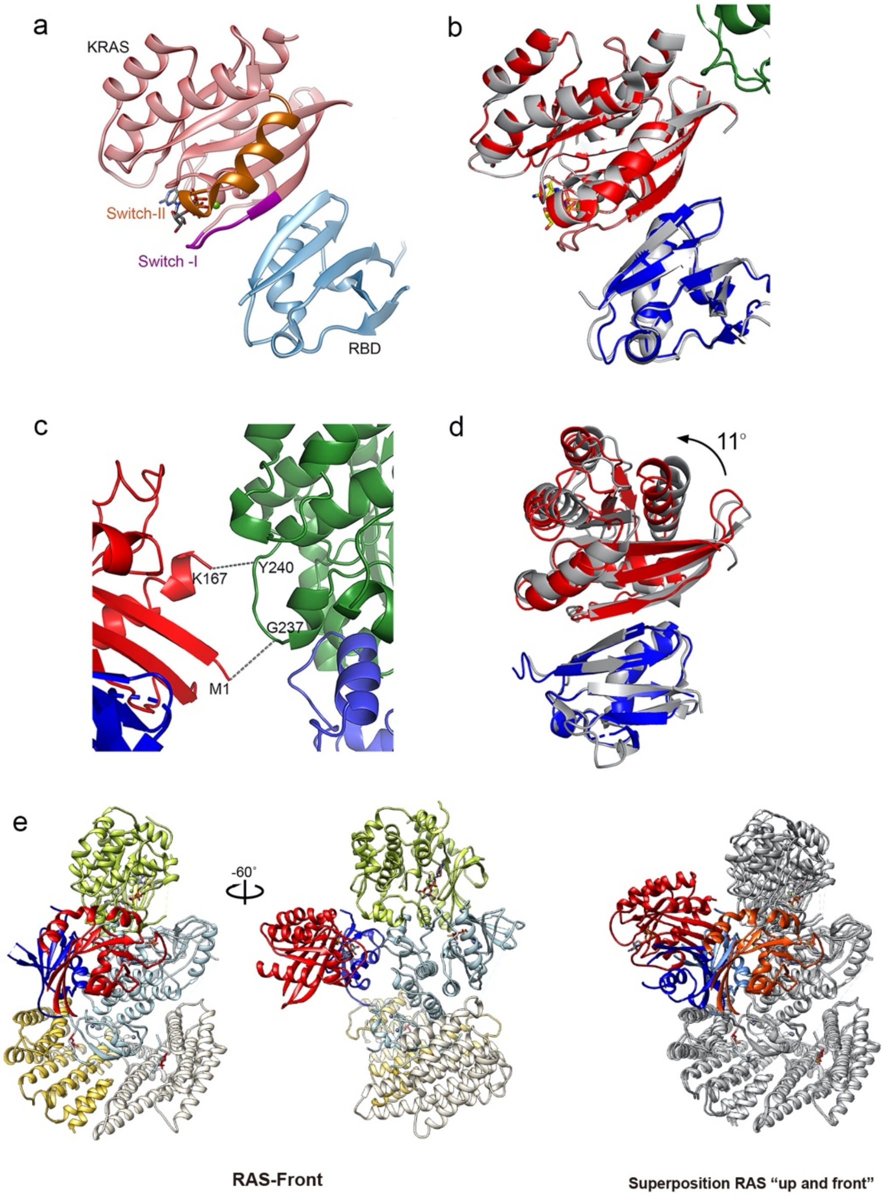

Supplementary Figure 2. Additional views and comparisons of the KRAS/RBD module. a, Ribbon diagram showing the interaction between KRAS and the BRAF RBD domain in the KRAS-up structure. The Switch-I and Switch-II regions of KRAS are highlighted in purple and orange, respectively. b, Superposition of the KRAS and RBD region of the KRAS-up structure with the previously determined structure of KRAS bound to the isolated RBD domain of CRAF (gray ribbon, PDB entry 6VJJ). c, Region of contact between KRAS (red) and MEK1 (green) in the KRAS-up structure. d, Superposition of the KRAS and RBD region of the KRAS-front structure with the previously determined structure of KRAS bound to the isolated RBD domain of CRAF (color-coded as in panel b). Superposition based on the RBD domains reveals a change in the relative orientation of KRAS by approximately 11°. e, Two views of the KRAS-front structure show the contact of the KRAS/RBD module at the interface between the BRAF and MEK1 kinase domains. Superposition of the KRAS-up and -front structures (right panel) reveals a ~165° difference in the relative orientation of the KRAS/RBD module.

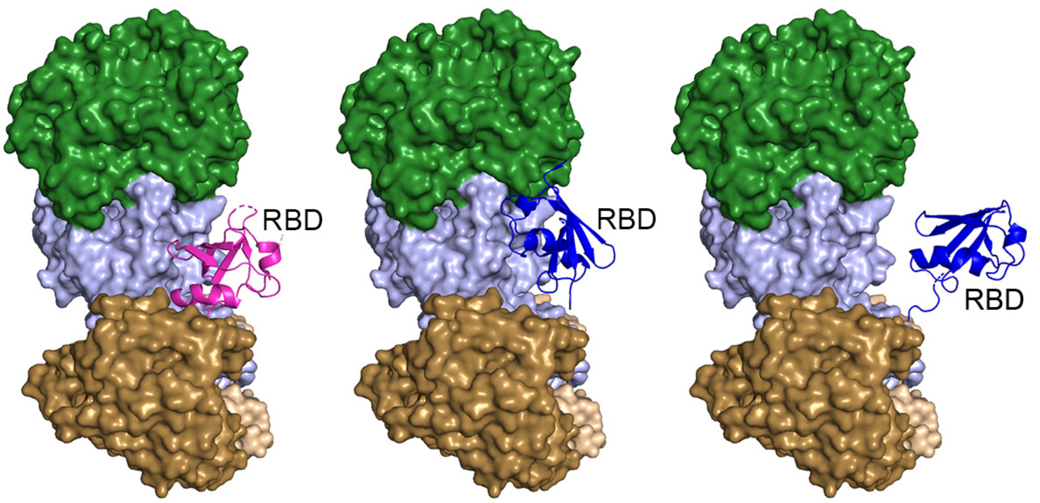

Supplementary Figure 3. Positions of the BRAF RBD domain in the free and KRAS-bound states. In each panel, the surface of the autoinhibited complex is shown with the 14-3-3 domain in tan, BRAF in blue, and MEK1 in green, and the RBD is shown in ribbon form. The autoinhibited structure reported by Martinez Fiesco et al. (PDB ID 7MFD) is shown on the left, with the RBD domain in magenta and the RAS-front and RAS-up structures reported here are shown in the center and right panels, respectively, with the RBD domain in dark blue. Note the distinct orientations and positions of the RBD domain in each structure. The general position of the RBD in the 7MFD structure corresponds to the poorly defined RBD density we previously observed in our autoinhibited structure and in the Δ155-BRAF/MEK1/14-3-3 structure reported here (see Supplementary Figure 5a below).

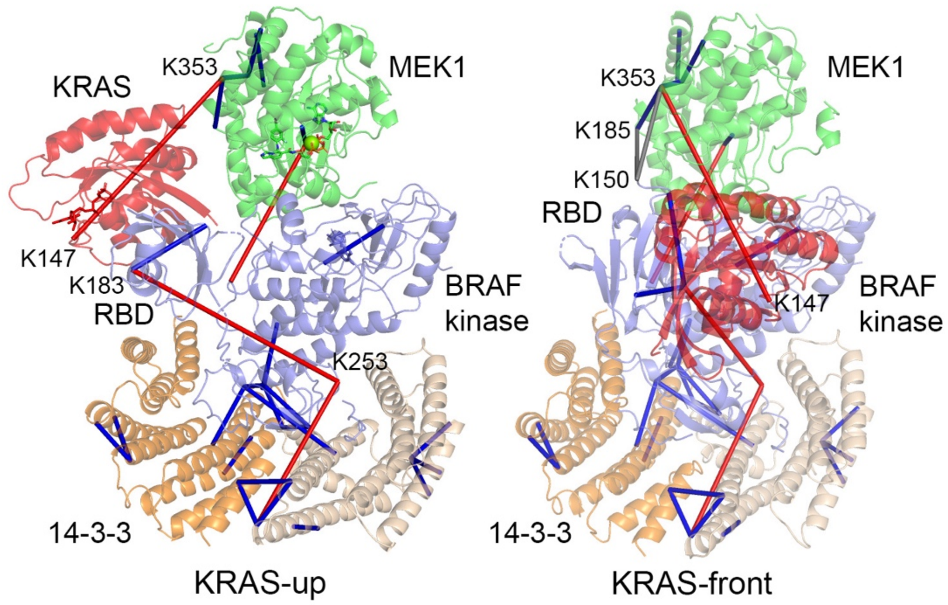

Supplementary Figure 4. Crosslinking mass spectrometry analysis of the BS3-crosslinked KRAS/BRAF/MEK1/14-3-3 sample. Crosslinks identified by mass spectrometry analysis of the BS3-treated sample are mapped onto the KRAS-up (left) and KRAS-front (right) structures. Residue numbers are given for inter-domain crosslinks to KRAS and the RBD domain. Crosslinks that are consistent with the structures (Ca to Ca distance of 20Å or less) are shown in dark blue, those that exceed this cutoff are shown in red. Two crosslinks between BRAF residue K150, which is just N-terminal to the RBD domain, and MEK1 residues K185 and K353 are indicated in gray and are consistent with the KRAS-front conformation. Source data are provided in the Source Data File.

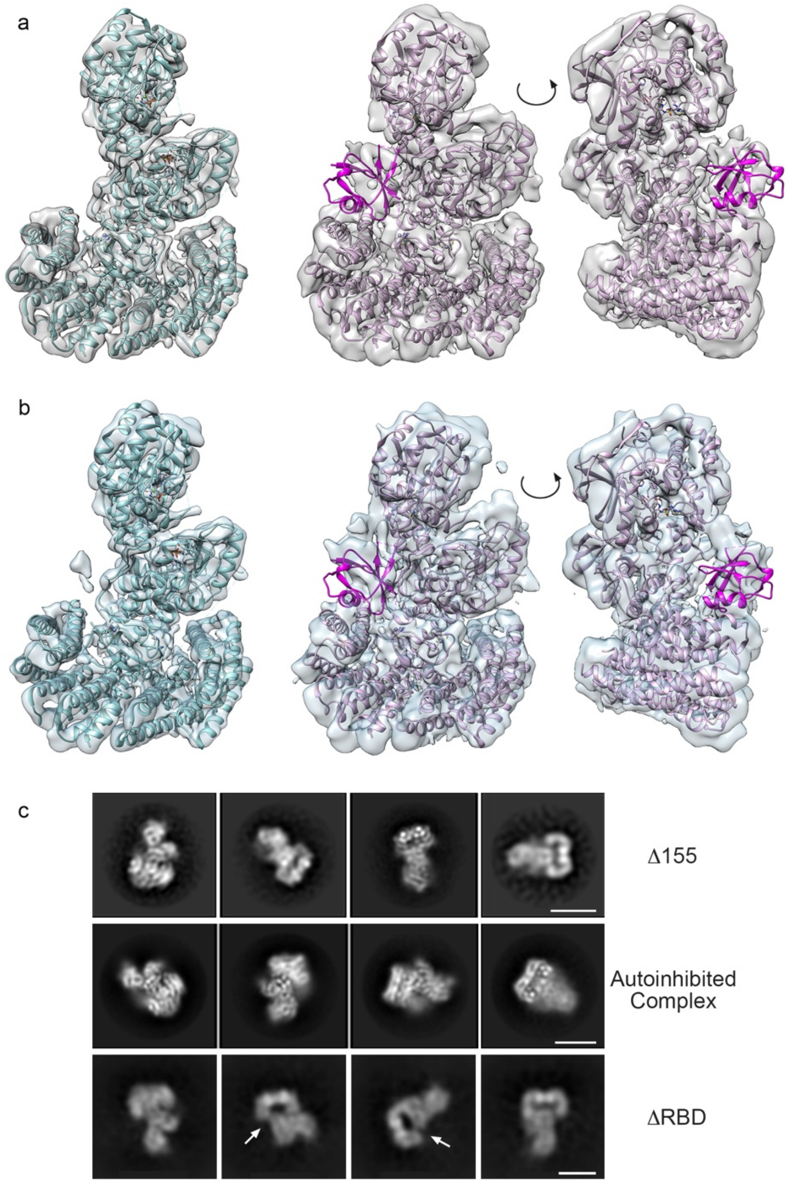

Supplementary Figure 5. Cryo EM imaging of Δ155- and ΔRBD-BRAF/MEK1/14-3-3 complexes in the autoinhibited state. a, Cryo EM density map from the 4.1Å single particle reconstruction of Δ155-BRAF/MEK1/14-3-3 at two contour levels. In the left panel, our prior structure of the autoinhibited full-length BRAF/MEK1/14-3-3 complex (PDB ID 6NYB) is fit to the map. In the center and right panels, the autoinhibited structure reported by Martinez Fiesco et al. (PDB 7MFD) is fit to the map, which is shown at a lower contour level in two orientations. Note the presence of additional density in the region corresponding to the RBD domain (magenta) at the lower contour level. b, As in panel a, but with our prior cryo EM density map of the full-length autoinhibited BRAF/MEK1/14-3-3 complex, filtered to 5Å resolution (Park et al., Nature 2019). Note that this map also contains additional density in the region corresponding to the RBD domain (magenta) at the lower contour level. c, Comparison of 2D class averages for the Δ155-, ΔRBD-, and full-length (autoinhibited) BRAF/MEK1/14-3-3 complexes. In the ΔRBD classes, note the presence of classes corresponding to those for the full-length autoinhibited complex. Also note the additional classes that appear to be partially open, with a lucency in the region of the 14-3-3 cradle that is occupied by the CRD domain in the fully autoinhibited state (arrows).

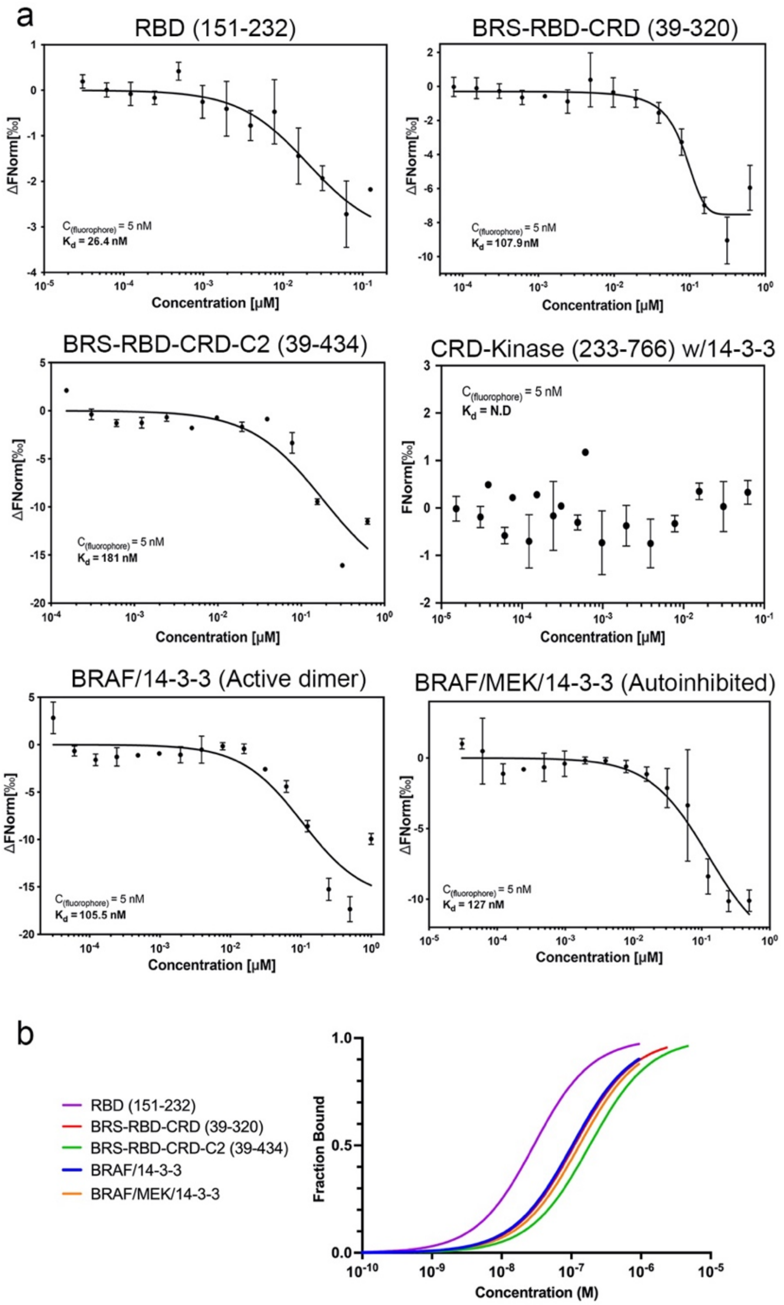

Supplementary Figure 6. Microscale thermophoresis studies of binding of KRAS to BRAF fragments and complexes. a, Microscale thermophoresis titration curves used to determine the affinity (dissociation constant, K_d) of GMP-PNP loaded KRAS^{GTPase} for the indicated BRAF fragments or for full-length BRAF bound to 14-3-3 in the active, dimeric state or full-length BRAF/MEK1/14-3-3 in the autoinhibited state, as reported in Table 1 of the main text. Error bars represent standard deviations from averages; experiments were performed three times, with independent dilutions for each (except for the CRD- kinase construct, for which no binding was observed in two independent experiments). b, Re-analysis of the titration curves above to show fraction bound as a function of concentration of the indicated BRAF protein or complex. Note that with the exception of the isolated RBD domain, affinities are not significantly different for the various BRAF proteins and complexes. Source data are provided in the Source Data File.

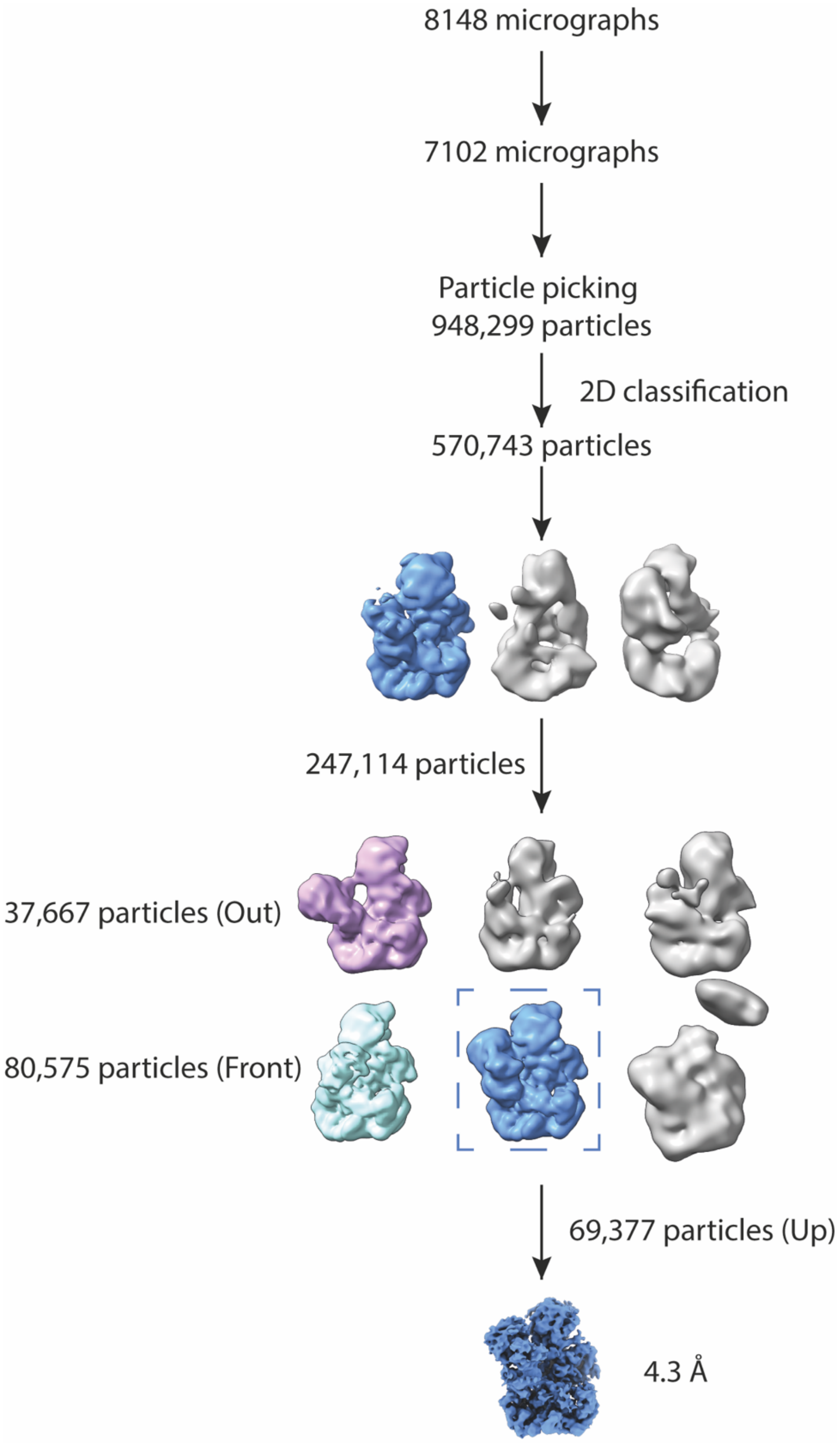

Supplementary Figure 7. Flowchart for single particle reconstruction of the KRAS/BRAF/MEK1/14-3-3 structure in the "KRAS-up" conformation (non-crosslinked). Three-dimensional classification identified particles in the KRAS-front conformation (cyan) and a KRAS-out conformation (pink) in addition the final "KRAS-up" conformation (blue).

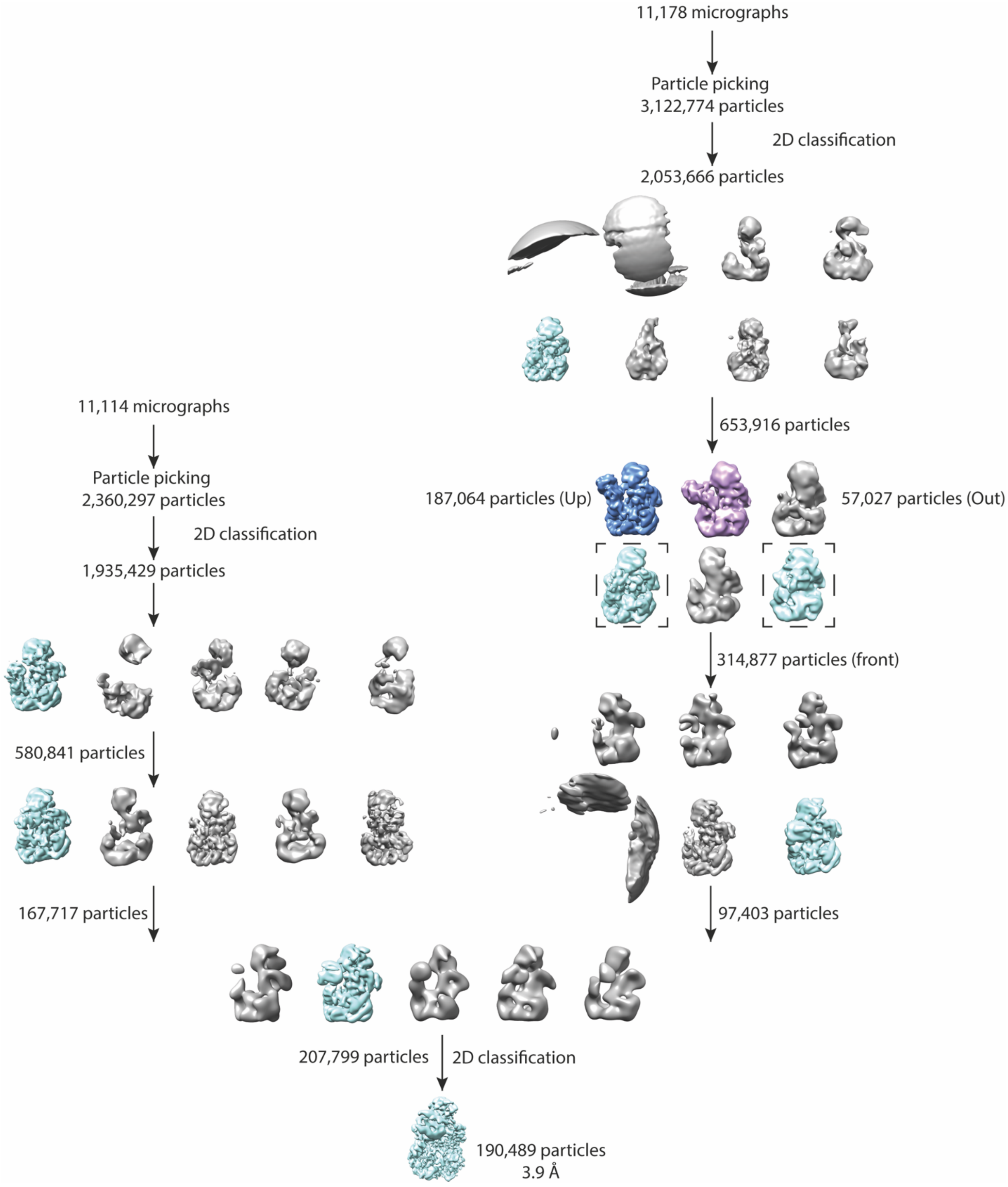

Supplementary Figure 8. Data processing flowchart for the KRAS/BRAF/MEK1/14-3-3 structure in the "KRAS-front" conformation (BS3-crosslinked). Particle stacks were merged from data sets collected from two grids prepared from the same protein sample. Three-dimensional classification identified particles in the "KRAS-up" conformation (blue) and a KRAS-out conformation (pink) in addition the final "KRAS-front" conformation (cyan).

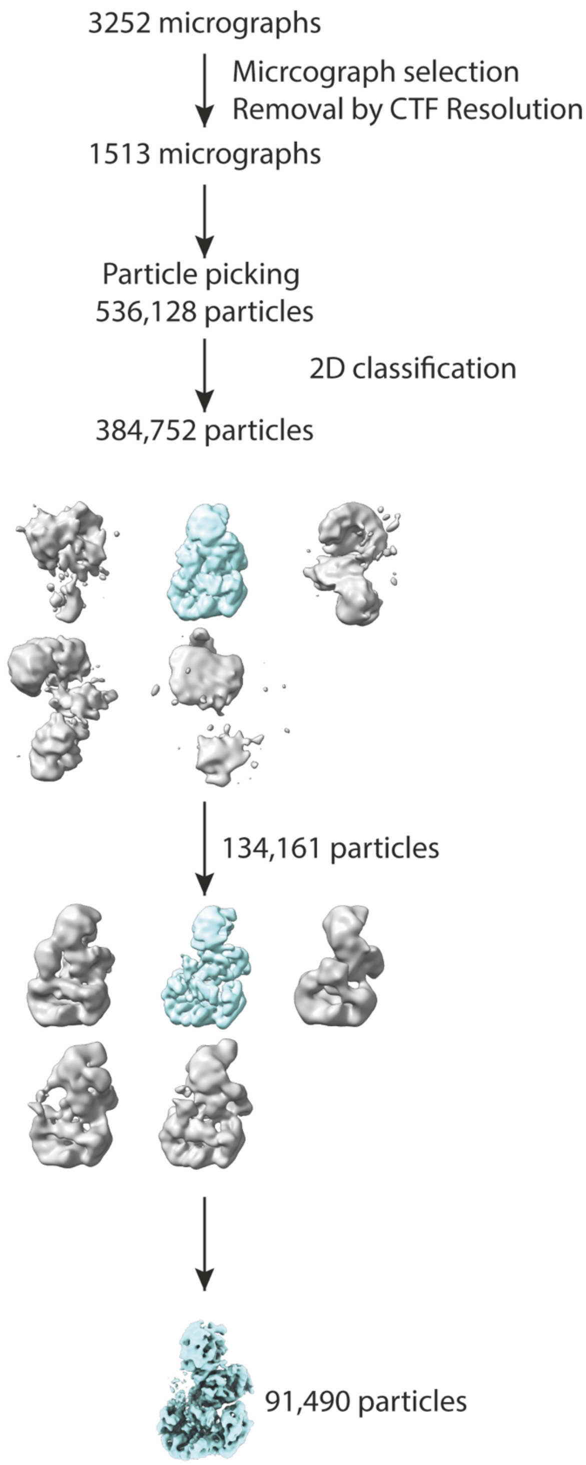

Supplementary Figure 9. Flowchart for single particle reconstruction of the Δ155-BRAF/MEK1/14-3-3 autoinhibited complex. The final reconstruction with 91,490 particles yielded a map at a resolution of 4.1Å, as illustrated in Supplementary Figure 5 above.

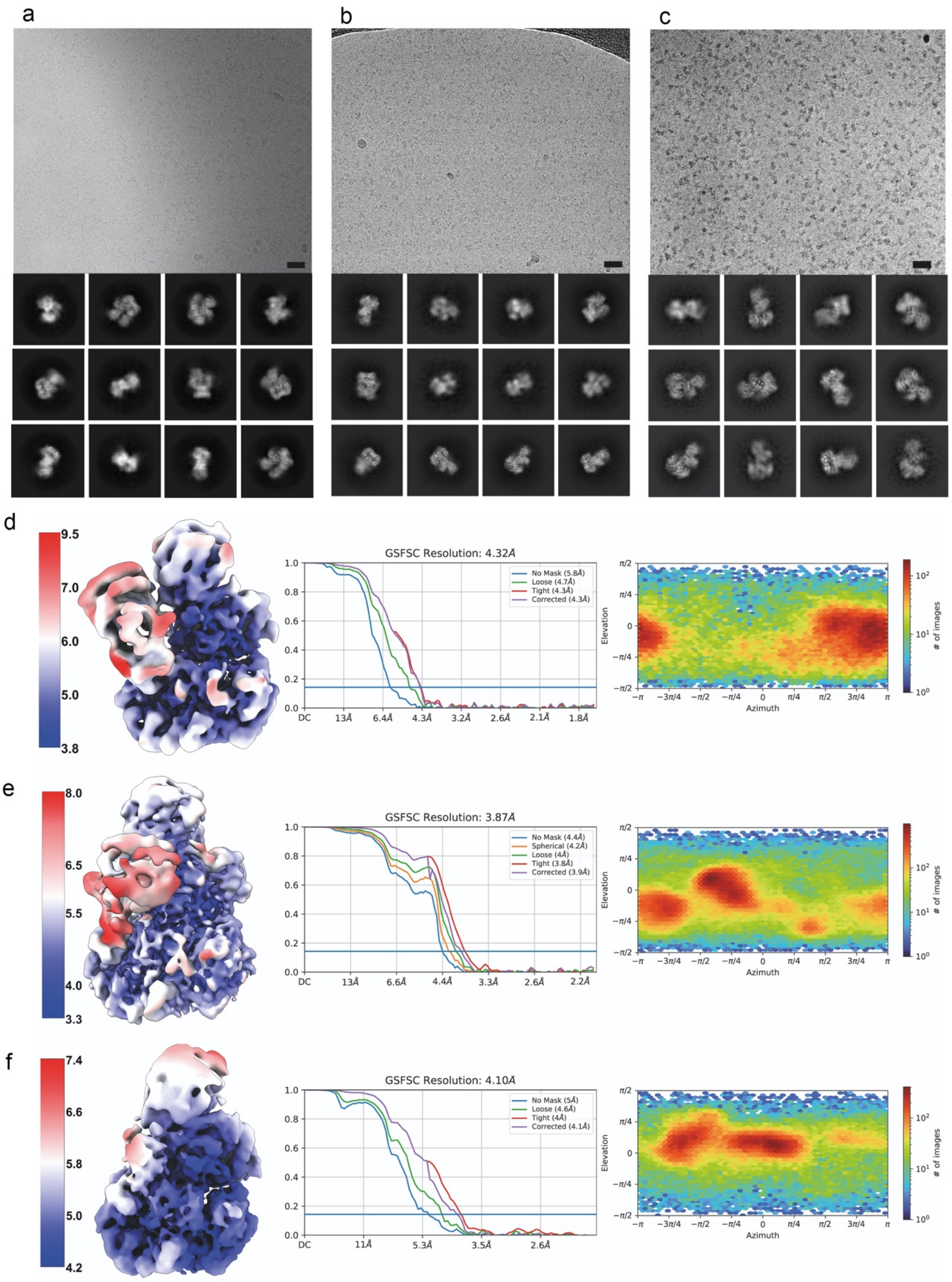

Supplementary Figure 10. Supporting information for cryo-EM structure determination. a-c, Section of a representative micrograph and 2D class averages from non-crosslinked, BS3-crosslinked, and Δ155-

BRAF/MEK1/14-3-3 samples, respectively. Scale bar is 25 nm. d-f, Cryo-EM density maps colored by resolution for the KRAS-up, KRAS-front, and Δ155-BRAF/MEK1/14-3-3 structures, respectively, together with corresponding gold-standard Fourier shell correlation curves and heatmaps showing distribution of particle orientations. For panels a and d, two separate data sets were acquired from two grids of the same sample and the data set with more images (8148) was chosen for single particle reconstruction. For panels b and e, two data collections of 11178 and 11144 images (from two grids prepared from the same sample) were merged for the analysis. For panels c and f, data were obtained from a single grid.

Supplementary References

1. Park, E. et al. Architecture of autoinhibited and active BRAF-MEK1-14-3-3 complexes. *Nature* **575**, 545-550 (2019).
2. Martinez Fiesco, J.A., Durrant, D.E., Morrison, D.K. & Zhang, P. Structural insights into the BRAF monomer-to-dimer transition mediated by RAS binding. *Nat Commun* **13**, 486 (2022).
