# Supplementary material for: Cryo-EM structure of a RAS/RAF recruitment complex
Source: Nat Commun. 2023 Jul 29;14:4580. doi: 10.1038/s41467-023-40299-6 (PMC10387098; doi:10.1038/s41467-023-40299-6)
Supplement: Supplementary file 3 — Description of Additional Supplementary Files [file 41467_2023_40299_MOESM3_ESM.docx]

**Supplementary Video Legends**

**Supplementary Video 1. Cryo EM density in the region of the KRAS/RBD module in the “KRAS-up” structure.** The RBD domain is shown in blue and KRAS in red.

**Supplementary Video 2. Cryo EM density in the region of the KRAS/RBD module in the “KRAS-front” structure.** The RBD domain is shown in blue and KRAS in red.

**Supplementary Video 3. Three-dimensional variability analysis of the KRAS/BRAF/MEK/14-3-3 complex.** Note that the density for the KRAS/RBD region largely fades out between the KRAS-up and KRAS-front positions, suggesting a lack of particles with intermediate conformations.
