# Supplementary material for: Cryo-EM structure of a RAS/RAF recruitment complex
Source: Nat Commun. 2023 Jul 29;14:4580. doi: 10.1038/s41467-023-40299-6 (PMC10387098; doi:10.1038/s41467-023-40299-6)
Supplement: Supplementary file 7 — Reporting Summary [file 41467_2023_40299_MOESM7_ESM.pdf]

nature portfolio

Corresponding author(s): MIchael J. Eck, Hyesung Jeon

Last updated by author(s): Jul 10, 2023

Reporting Summary

Nature Portfolio wishes to improve the reproducibility of the work that we publish. This form provides structure for consistency and transparency in reporting. For further information on Nature Portfolio policies, see our Editorial Policies and the Editorial Policy Checklist.

Statistics

For all statistical analyses, confirm that the following items are present in the figure legend, table legend, main text, or Methods section.

n/a	Confirmed	
[ ]	[x]	The exact sample size (*n*) for each experimental group/condition, given as a discrete number and unit of measurement
[ ]	[x]	A statement on whether measurements were taken from distinct samples or whether the same sample was measured repeatedly
[x]	[ ]	The statistical test(s) used AND whether they are one- or two-sided<br>*Only common tests should be described solely by name; describe more complex techniques in the Methods section.*
[x]	[ ]	A description of all covariates tested
[x]	[ ]	A description of any assumptions or corrections, such as tests of normality and adjustment for multiple comparisons
[ ]	[x]	A full description of the statistical parameters including central tendency (e.g. means) or other basic estimates (e.g. regression coefficient) AND variation (e.g. standard deviation) or associated estimates of uncertainty (e.g. confidence intervals)
[x]	[ ]	For null hypothesis testing, the test statistic (e.g. *F*, *t*, *r*) with confidence intervals, effect sizes, degrees of freedom and *P* value noted<br>*Give P values as exact values whenever suitable.*
[x]	[ ]	For Bayesian analysis, information on the choice of priors and Markov chain Monte Carlo settings
[x]	[ ]	For hierarchical and complex designs, identification of the appropriate level for tests and full reporting of outcomes
[x]	[ ]	Estimates of effect sizes (e.g. Cohen's *d*, Pearson's *r*), indicating how they were calculated

Our web collection on statistics for biologists contains articles on many of the points above.

Software and code

Policy information about availability of computer code

Data collection	Standard widely available software was used for, including SerialEM for Krios microscope and K2, K3 detector
Data analysis	Standard widely available software was used for structure determination, including RELION3 & 4 , crYOLO (ver.1.9.3), Coot (ver.0.9.8.7) and PHENIX (ver. 1.20.1-4487). References are provided in the References section.

For manuscripts utilizing custom algorithms or software that are central to the research but not yet described in published literature, software must be made available to editors and reviewers. We strongly encourage code deposition in a community repository (e.g. GitHub). See the Nature Portfolio guidelines for submitting code & software for further information.

Data

Policy information about availability of data

All manuscripts must include a data availability statement. This statement should provide the following information, where applicable:

- Accession codes, unique identifiers, or web links for publicly available datasets
- A description of any restrictions on data availability
- For clinical datasets or third party data, please ensure that the statement adheres to our policy

The cryo-EM MAP and atomic coordinates have been deposited with the EMDB and PDB, respectively (EMD-27428, EMD-27429, EMD-40253, PDB: 8DGS, 8DGT). The mass spectrometry data are depositied in PRIDE (PXD042584). All links are reported in the manuscript Data Availibility section including previously reported PDB (6NYB, 6VJJ, 7MFD) used in this study.

Research involving human participants, their data, or biological material

Policy information about studies with human participants or human data. See also policy information about sex, gender (identity/presentation), and sexual orientation and race, ethnicity and racism.

Reporting on sex and gender	Not applicable
Reporting on race, ethnicity, or other socially relevant groupings	Not applicable
Population characteristics	Not applicable
Recruitment	Not applicable
Ethics oversight	Not applicable

Note that full information on the approval of the study protocol must also be provided in the manuscript.

Field-specific reporting

Please select the one below that is the best fit for your research. If you are not sure, read the appropriate sections before making your selection.

[x] Life sciences [ ] Behavioural & social sciences [ ] Ecological, evolutionary & environmental sciences

For a reference copy of the document with all sections, see nature.com/documents/nr-reporting-summary-flat.pdf

Life sciences study design

All studies must disclose on these points even when the disclosure is negative.

Sample size	SIngle particle reconstruction was performed from one (for non-crosslinked sample, Del155-BRAF complex and Del-RBD-BRAF complex) or two (for crosslinked sample) data sets. One data set from one grid was sufficient to determine the structure, but we tried two data sets combined for crosslinked sample in order to improve resolution.
Data exclusions	No excluded data.
Replication	The experiments regarding the Figure1b, c and Figure 3 are performed at least three times. The experiment for Supplementary Figure 4 was performed once, and three times for Supplementary Figure 6. The number of repetition for Supplementary Figure 10 is same as sample size above and mentioned in the Legend and Methods.
Randomization	Randomization is not neccessary or appropriate in these biochemical and biophysical experiments.
Blinding	Blinding is not neccessary or appropriate in these biochemical and biophysical experiments.

Reporting for specific materials, systems and methods

We require information from authors about some types of materials, experimental systems and methods used in many studies. Here, indicate whether each material, system or method listed is relevant to your study. If you are not sure if a list item applies to your research, read the appropriate section before selecting a response.

Materials & experimental systems

n/a	Involved in the study	
[ ]	[x]	Antibodies
[ ]	[x]	Eukaryotic cell lines
[x]	[ ]	Palaeontology and archaeology
[x]	[ ]	Animals and other organisms
[x]	[ ]	Clinical data
[x]	[ ]	Dual use research of concern
[x]	[ ]	Plants

Methods

n/a	Involved in the study	
[x]	[ ]	ChIP-seq
[x]	[ ]	Flow cytometry
[x]	[ ]	MRI-based neuroimaging

Antibodies

Antibodies used	Anti-ERK (Cell signaling technology, #9102) RRID:AB_330744, 1:1000 dilution<br>Anti-pERK (Cell signaling technology, #45899, (D13.14.4E)) RRID:AB_2315112, 1:1000 dilution

Validation	Listed RRID for each antibody above.

Eukaryotic cell lines

Policy information about cell lines and Sex and Gender in Research

Cell line source(s)	Gibco™ Sf9 cells in Sf-900™ II SFM (ThermoFisher Scientific, Cat.no.11496015)
Authentication	Cells were purchased from ThermoFisher and not independently authenticated.
Mycoplasma contamination	Vendor (ThermoFisher)'s product quality confirms negative.
Commonly misidentified lines (See ICLAC register)	Not applicable
