# Supplementary figures and images for: Cryo-EM structure of a RAS/RAF recruitment complex
Source: Nat Commun. 2023 Jul 29;14:4580. doi: 10.1038/s41467-023-40299-6 (PMC10387098; doi:10.1038/s41467-023-40299-6)

**a**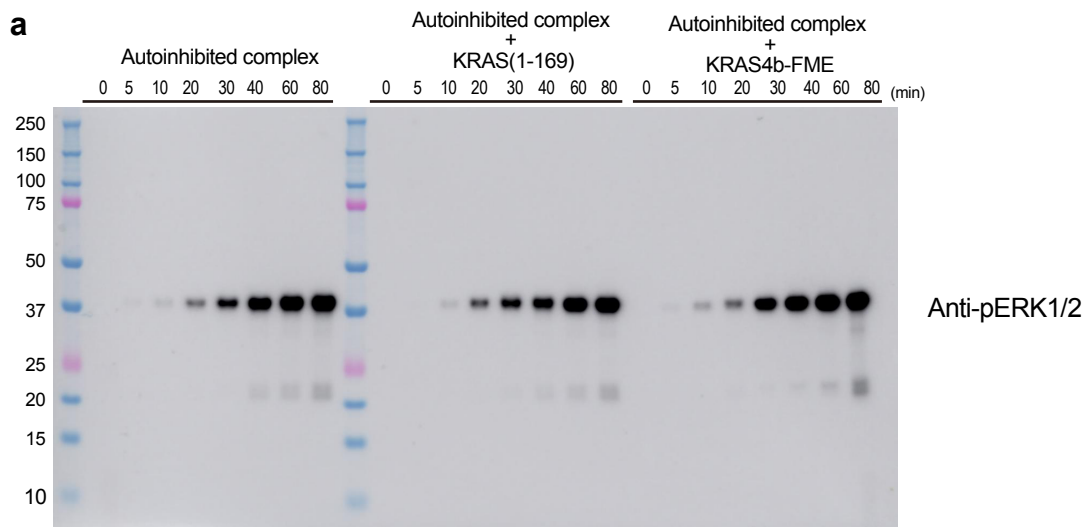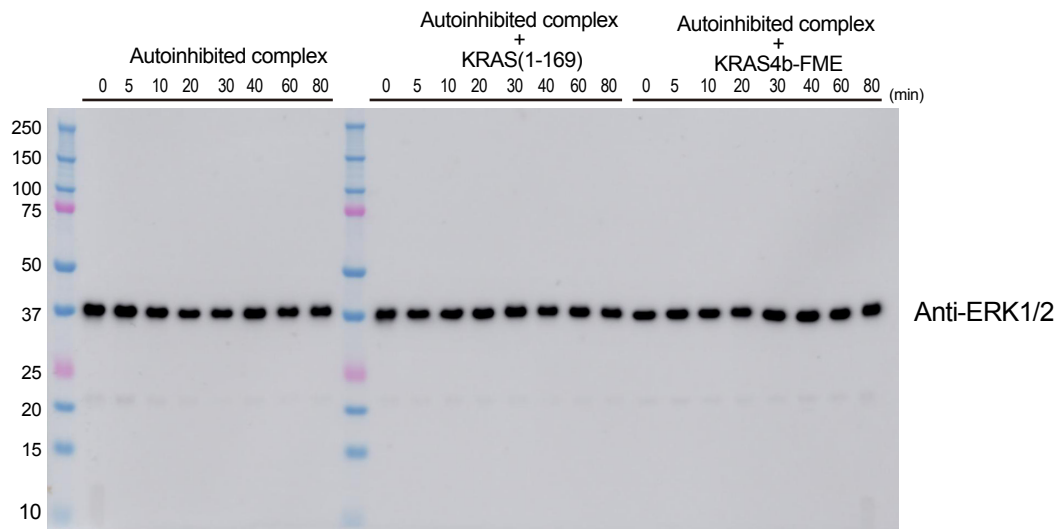

**b**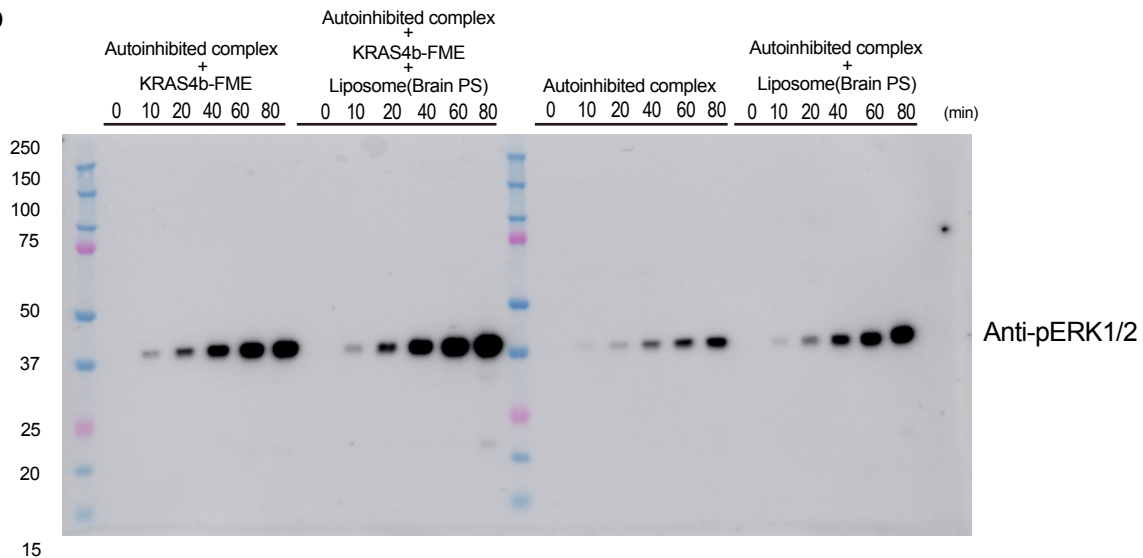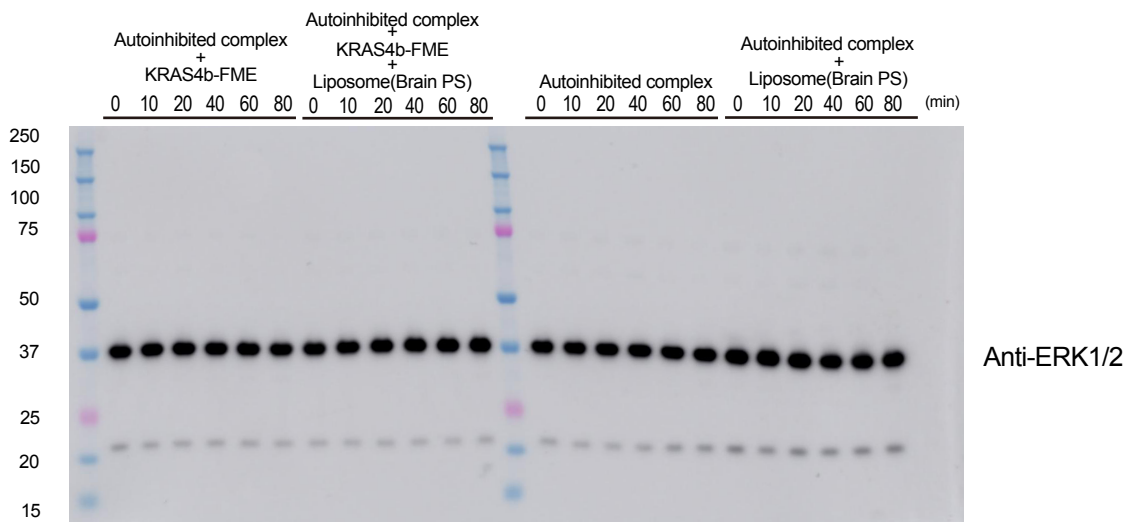

**c**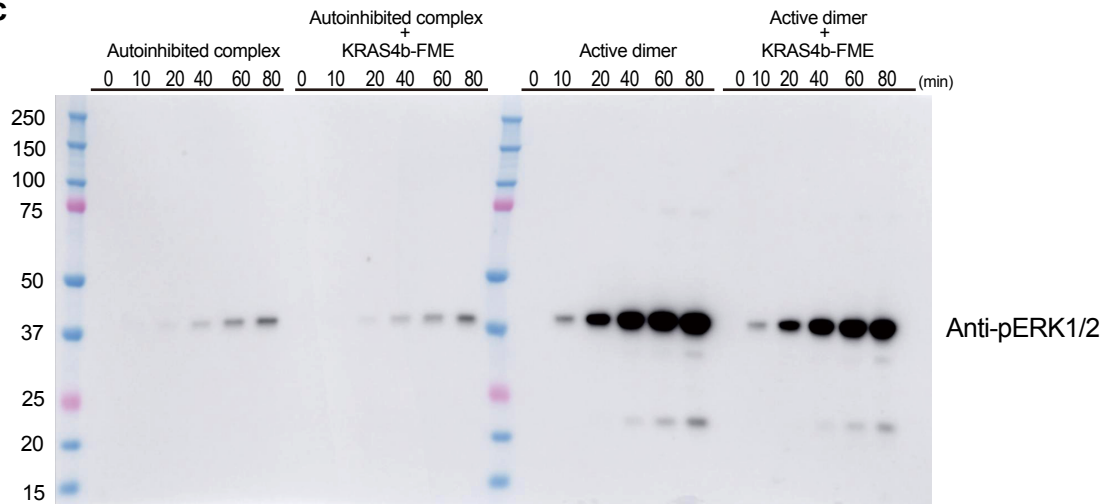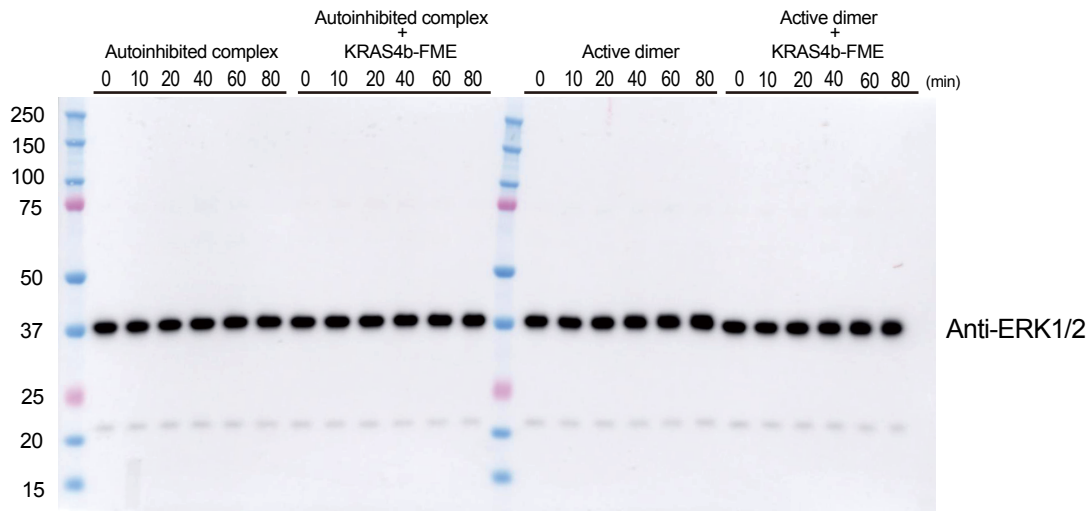

Supplement: Supplementary file 8 — Source Data [file 41467_2023_40299_MOESM8_ESM.zip › Fig3_annotated-blots.pdf]
